# Mixed and non-competitive enzyme inhibition: underlying mechanisms and mechanistic irrelevance of the formal two-site model

**DOI:** 10.1080/14756366.2023.2245168

**Published:** 2023-08-14

**Authors:** Alessandro Pesaresi

**Affiliations:** Istituto di Cristallografia – Consiglio Nazionale delle Ricerche, Trieste, Italy

**Keywords:** Mixed inhibition, non-competitive inhibition, inhibition mechanism

## Abstract

The formal mechanism of linear mixed and non-competitive enzyme inhibition implies the binding of inhibitors to both the active site of the free enzyme in competition with the substrate, and to an allosteric site on the enzyme-substrate complex. However, it is evident from a review of the scientific literature that the two-site mechanism is frequently mistaken as the actual underlying mechanism of mixed inhibition. In this study, we conducted a comprehensive assessment of the mechanistic relevance of this type of inhibition using a statistical approach. By combining a statistical analysis of the inhibition cases documented in the BRENDA database with a theoretical investigation of inhibition models, we conclude that mixed inhibitors exclusively bind to the active site of enzymes. Hence ruling out any implication of allosteric sites and depriving the two-site model of any mechanistic relevance.

## Introduction

Prompted to “write a definition of mixed-type enzyme inhibition”, ChatGPT elaborated the following composition:

Mixed-type enzyme inhibition is a form of enzyme inhibition where the inhibitor can bind to both the free enzyme and the enzyme-substrate complex, resulting in distinct effects on both the enzyme’s catalytic activity and its affinity for the substrate. In mixed-type inhibition, the inhibitor can interact with the enzyme at either the active or a separate allosteric site. […]

Mixed-type enzyme inhibition is a complex regulatory mechanism that can have important implications in various biological processes and the development of therapeutic strategies targeting enzyme activity. Understanding the nature of mixed-type inhibition is crucial for studying enzyme kinetics, drug discovery, and the design of effective enzyme inhibitors.

Because the algorithms of ChatGPT have been trained using a massive amount of text data, the response it provided can be considered as a representative description of mixed inhibition that aligns with the general understanding presented in articles, books and other scientific publications.

Also, it seems safe to assume that most biochemists, drug designers and, in general, most of those who concern themselves with enzyme inhibition, would substantially agree with this definition.

**Figure 1.**
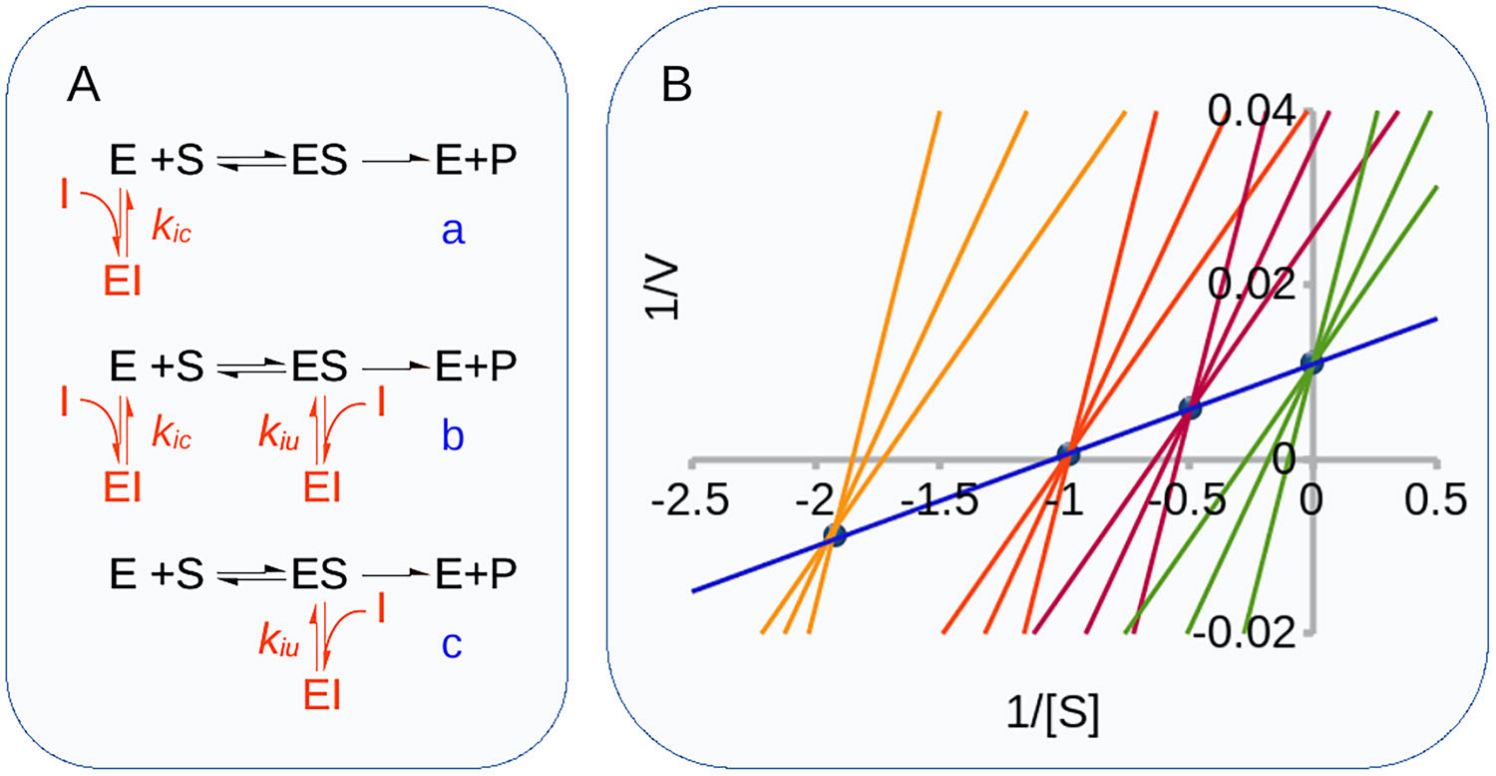
Inhibition mechanisms and patterns. **(A)** Formal inhibition mechanisms. Competitive inhibition (a), mixed-type inhibition (b), uncompetitive inhibition (c). **(B)**. Inhibition pattern of competitive and mixed-type inhibition. In a 1/V versus 1/[S] plot of initial rates, competitive inhibitors generate a bundle of lines (green) that intercept the non-inhibited reaction trace (blue) on the 1/V axis. Mixed-type inhibitors reduce both *1/V* and *V/K_M_* by binding the free enzyme (E) and the ES complex. They generate bundles of lines that intercept on the left of the 1/V axis at 1/[S] values equal to -*K_IC_*/*K_IU_*. For pure non-competitive inhibitors (*K_IC_=K_IU_*, orange lines), the intercept is on the 1/[S] axis. If *K_IC_*<*K_IU_*, (red lines, *K_IC_*/*K_IU_=*0.5), the intercept is above the 1/[S] axis, and it is below if *K_IC_*>*K_IU_*, (yellow lines, *K_IC_*/*K_IU_=*2).

Yet, the above statement features a misleading inaccuracy: it is not true that mixed inhibitors necessarily bind to topologically distinct sites on the free enzyme and the enzyme-substrate complex. In defence of ChatGTP, it must be said that this error reflects a very common misconception about the significance of kinetic mechanisms. Reversible inhibition, in fact, is classified as competitive, mixed or uncompetitive, on the basis of the effect that the inhibitor exerts on *K_cat_* and *K_cat_*/*K_m_*, which classically is measured as an intercept and/or a slope effect of a double-reciprocal plot, respectively (Figure 1). One aspect that is often overlooked however, is that there is no bijective relation between the type of inhibition, whose definition is only operational, and the underlying molecular mechanisms^1^. The steady-state analysis of initial velocities can never establish a molecular mechanism beyond doubt, therefore, it is critical to point out that the three formal mechanisms of inhibition (Figure 1(A)) only represent the simplest mechanisms that are consistent with the experimental observations^1^.

This means that the type of inhibition cannot be taken as a sufficient proof of any given molecular mechanism. For example, although it is true that competitive inhibition is generally caused by inhibitors that, in agreement with the conventional mechanism (Figure 1(A)) bind the active site in competition with the substrate, exceptions where competition results from the binding of allosteric sites have also been reported^2^^,^^3^. In contrast, only on rare occasions do uncompetitive inhibitors actually bind the ES complex^4^^,^^5^. The most common sources of uncompetitive inhibition being, by far, reactions with more than one substrate with compulsory-order mechanisms^6^^,^^7^ where the inhibitor binds to the binding site of the constant substrate.

With mixed and pure non-competitive inhibition the picture is even more complex and tricky: the conventional mechanism, reported in all textbooks and acknowledged by ChatGPT (Figure 1(A)), implies the unlikely event of one inhibitor binding, with somewhat similar affinity, to two distinct sites, i.e. the active site and an allosteric site of the ES complex.

Seasoned enzymologists are well aware that this two-site model is mainly just a convenient formal way to define mixed inhibition and that it should not be intended as a real molecular mechanism.

Nevertheless, general biochemistry textbooks usually do not go beyond this description, ingraining the wrong belief that mixed and non-competitive inhibitors actually act by binding the active site and a topologically distinct allosteric site. Sometimes, even educational research papers^8^^,^^9^ and enzymology manuals^10^^,^^11^ indulge in the same oversimplification.

Resorting to authoritative sources certainly reduces the risk of misapprehension, but for those who do not have a strong background in enzymology, attaining a proper understanding of mixed inhibition and its molecular determinants remains arduous. For example, Cornish-Bowden in “Fundamental of Enzyme Kinetics” clearly explains the limits of the formal two-site mechanism and suggests that mixed inhibition occurs mainly as a case of product inhibition with iso-mechanism enzymes^12^. Johnson, in “Kinetic Analysis for the New Enzymology”, adds that mixed inhibition, analogous to uncompetitive inhibition, occurs mainly with multi-substrate reactions, either as a form of product inhibition or, for ternary-complex mechanisms, when a dead-end inhibitor binds before the variable substrate.^7^ Copeland instead, in “Evaluation of Enzyme Inhibition in Drug Discovery”, mentions five mechanisms through which active site-directed inhibitors can cause inhibition patterns typical of mixed inhibition.^3^ However, these five mechanisms are treated as exceptions to a general rule. With the general rule being, again, the formal two-site mechanism.

As this brief example shows, the picture that emerges is so complicated and confusing that, as a matter of fact, many authors just rely on the widely acknowledged idea that the inhibition is of the mixed-type if, and only if, the inhibitor binds both the active site of the free enzyme and an allosteric site of the enzyme-substrate complex.

We conducted a brief survey by searching Google Scholar with the query “mixed inhibition mechanism”. It turned out that in 16 out of the first 30 papers that report cases of mixed inhibition, the authors explicitly assumed the two-site model as the actual underlying mechanism^13–28^. It is worth noting that this misconception is common in medium-low tier journals as well as in high impact ones^29–33^.

Often, especially in drug design projects, the ultimate objective of kinetic analysis is to elucidate a molecular mechanism for the obvious reason that it can help to rationalise the improvement of lead compounds. The belief that mixed inhibition implies the binding of an active and an allosteric site is so deeply enrooted, however, that many researchers just take it as consolidated truth with no need for further investigations. On the other hand, establishing the actual inhibition mechanism would require thorough biochemical or biophysical studies that are not always within the scope of those works. Therefore, there is a question, of fundamental importance for medicinal chemists and drug designers, that still stands: to what extent, if any, does the two-site mechanism account for the reported cases of mixed-type and non-competitive inhibition?

Here, we try to provide an answer to that one question employing a statistical approach: forty years ago, Cornish-Bowden noted that uncompetitive inhibition almost always occurs in the form of mixed inhibition and that in mixed inhibition, the uncompetitive component never prevails over the competitive counterpart (i.e. *K_ic_*≤*K_iu_*), ^34^. This intriguing anomaly - it would imply that mixed inhibitors bind the active site always more effectively than the allosteric site - evidently do not find explanation in the framework of the two-site mechanism. Indeed, there are five main mechanisms, or circumstances, by which an active site-bound inhibitor might appear to be mixed-type or non-competitive when analysed by steady-state kinetics (four of which have been revised in reference^35^) In this study, we demonstrated that in the mixed inhibition caused by these five *mixed inhibition-mimicking* (MIM) mechanisms, *K_ic_* is necessarily always less than or equal to *K_iu_*.

The combination of this latter finding with the results of a statistical analysis, which confirmed the overwhelming preponderance of the competitive component of mixed inhibition, strongly suggests that the five MIM mechanisms are the actual underlying mechanisms of mixed-type inhibition, and hence, that, with virtually no exception, mixed inhibitors bind only the substrate site of the free enzyme. This excludes the implication of any interaction with any allosteric site and, in conclusion, points to a substantial mechanistic irrelevance of the formal two-site model of mixed inhibition.

## Methods

### Simulation of mixed inhibition-mimicking mechanisms

To simulate MIM mechanisms, initial velocities were generated using KinTek Explorer^36^^,^^37^, and the inhibition constants *K_ic_* and *K_iu_* were determined by the Dixon and Cornish-Bowden plots, respectively. Velocities were detected 60 s after the start of the reactions. For each mechanism, the reaction scheme, kinetic constants and concentration of reagents are given below.

#### Case I – tight binding inhibitors


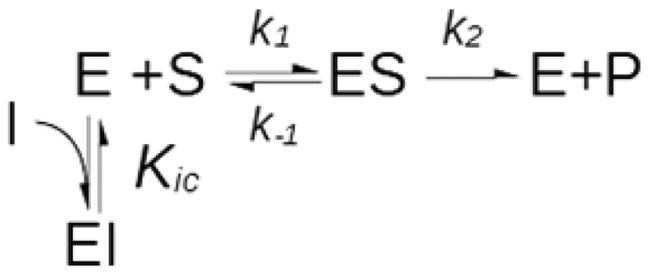


*k_1_* = 2·10^7^ mol^−1^s ^− 1^, *k_-1_* = 50 s ^− 1^, *k_2_* = 50s ^− 1^.

*K_ic_* = 0.01–10 nM.

[E] = 1 nM, [S] = 10–400 µM, [I] = 0–10 nM.

#### Case II – time dependent inhibition


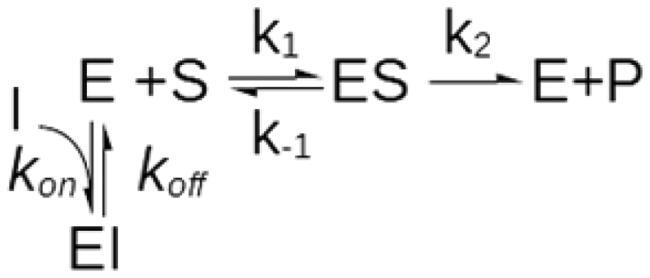


*k_1_* = 1·10^8^ mol^−1^s ^−1^, *k_-1_* = 1 s ^−1^, *k_2_* = 1000s ^−1^, *k_off_* = 1 0 ^−5^–10^3^ s ^−1^, *k_off_*/*k_on_* = 1 0 ^−6^ M.

[E] = 100 pM, [S] = 1–200 µM, [I] = 0.5–50 µM.

#### Case III – Multisubstrate reactions


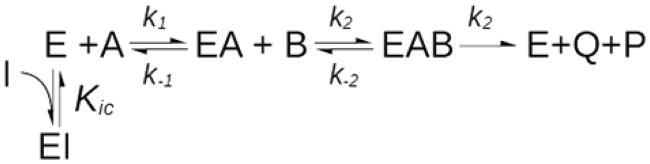


*k_1_* = 1·10^8^ mol^−1^s ^−1^, *k_-1_* = 2500s ^−1^, *k_2_* = 1·10^8^ mol^−1^s ^−1^, *k_-2_* = 400 s ^−1^, and *k_2_* = 100 s ^−1^.

[E] = 1 nM, [A] = 0.1–1000 µM, [B] = 2.5–200 µM, [I] = 0–10 nM.

*K_s_=25* µM, *K_M(A)_*=1 µM, *K_M(B)_*=5 µM, K_i_=10 nM, *k_ca_*_t_=100 s ^−1^, *K_cat_/k_-1_* = 25.

#### Case IV – iso-mechanisms


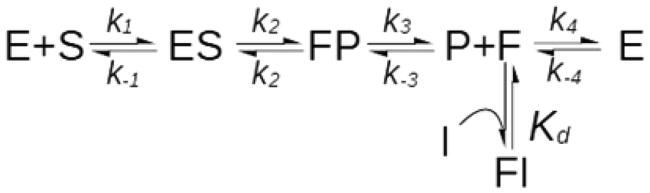


[E] = 0.1 nM, [S] = 2–100 µM, [I] = 0–100 nM, *K_d_*=10 nM.

*k_1_* = 10^8^ mol^−1^s ^−1^, *k_-1_* = 100 s ^−1^, *k_3_* = 100 mol^−1^s ^−1^, *k_-3_* = 10^8^ s ^−1^.

The combinations of the *k_2_*, *k_-2_*, *k_4_* and *k_-4_* kinetic constant values used to simulate the different F_iso_ values are given in Table 1.

**Table 1.** Combination of the *k_2_*, *k_-2_*, *k_4_* and *k_-4_* constants used to obtain the different F_iso_ values.

F_iso_	0	0.1	0.2	0.5	0.7	1
*k_2_* (mol^-1^s^-1^)	100	111	125	200	333	10^6^
*k_-2_* (s^-1^)	100	111	125	200	333	10^6^
*k_4_* (mol^-1^s^-1^)	10^6^	1000	500	200	143	100
*k_-4_* (s^-1^)	10^6^	1000	500	200	143	100

#### Case V – exo-site


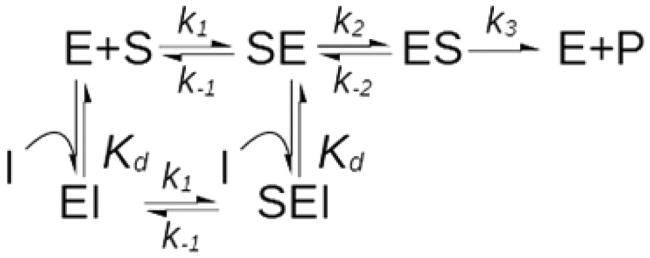


[E] = 0.1 nM, [S] = 0.2–100 µM, [I] = 0–100 nM, *K_d_* = 10 nM.

*k_1_* = 10^8^ mol^−1^s ^−1^, *k_-1_* = 1 s ^−1^, *k_2_* = 10^6^–50 s ^−1^, *k_-2_* = 1 s ^−1^, *k_3_* = 50–10^6^ s ^−1^.

## Results

### Statistics of enzyme inhibition

**Figure 2.**
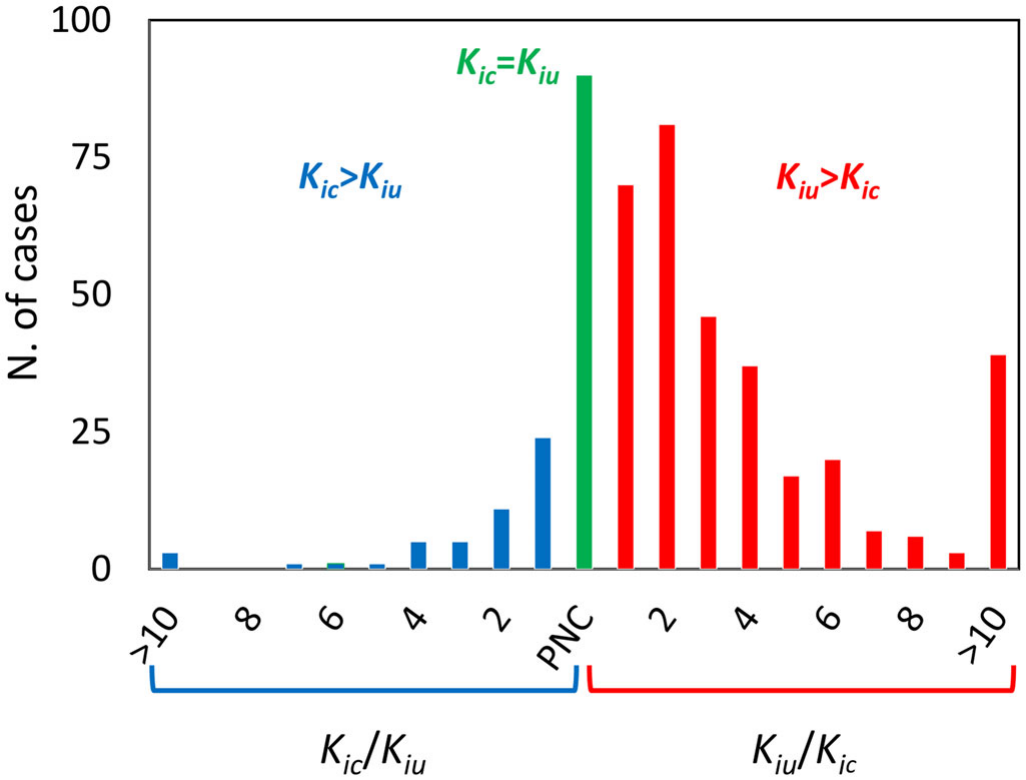
Distribution of the *K_iu_*-*K_ic_* ratio frequencies of mixed-type inhibitors. For inhibition dominated by the competitive component (*K_iu_*>*K_ic_*, red bars), the data are presented as *K_iu_*/*K_ic_*. Data of mixed-inhibitions with *K_ic_*>*K_iu_* (blue bars) are presented as *K_ic_*/*K_iu_* ratio. Pure non-competitive inhibition (PNC) is shown in green. The competitive component is prevalent in 70% of cases, pure non-competitive inhibition represents 20% of cases, while the dominance of the uncompetitive component is observed only in 10% of cases. The total number of cases analysed was 467.

The quantitative statistical analysis of the available kinetic data is a precondition to turn anecdotal evidence into more solid objective observations and hence to draw conclusions on the significance of mixed-inhibition. Unfortunately, none of the extant repositories stores information about the mechanism of inhibition in a structured and systematic way. In the BRENDA database^38^, for example, which contains entries for more than 220,000 enzyme inhibitors and 200,000 reference citations, this kind of information has only occasionally been reported by the database curators and can be found as scattered notes in the commentary fields of a minority of the entries. Because these entry notes are not searchable, to build a reliable statistic, the relevant data were mined from the downloadable BRENDA text file. A search in this almost five million-line document of the terms “competitive”, “noncompetitive”, “mixed” and “uncompetitive” inhibitors returned a total of 7563 hits. On the basis of the occurrence of each term and in agreement with what had already been reported^29^, competitive inhibition was the most common type of inhibition, accounting for 73% of total cases. Mixed/non-competitive and uncompetitive inhibition accounted for 22% and 5% of cases, respectively. To deepen the “*K_ic_* versus *K_iu_* issue”, the 464 entries containing the term “mixed” or “mixed-type” inhibition/inhibitor were further analysed. These entries point to 266 reference articles, from which it has been possible to retrieve 467 *K_ic_*-*K_iu_* couples (Figure 2). If mixed inhibition was caused by the binding of inhibitors to two distinct sites, the dominance of the competitive or of the uncompetitive component would be equally probable, and a symmetric and rather flat distribution of the *K_ic_*/*K_iu_* and *K_iu_*/*K_ic_* ratios around a central value of 1 would be expected. The analysis of these *K_ic_*-*K_iu_* values, however, showed that the dominance of the competitive component is overwhelmingly preponderant (*K_ic_*≤*K_iu_* in 90% of the cases) and that pure non-competitive inhibition (*K_ic_*=*K_iu_*) occurs with an unrealistically high frequency (*K_ic_*/*K_iu_*=1 in 20% of cases), (Figure 2).

It is worth noting that the large majority of the gathered *K_i_*s were originally obtained by the graphical analysis of initial velocity data, a method that is known to be rather inaccurate^39^^,^^40^. If to account for this inaccuracy some allowance is granted and inhibition is considered to be pure non-competitive up to a *K_ic_*/*K_iu_* ratio between 0.5 and 2 (rather than exactly equal to 1), then the frequency of pure non-competitive inhibition jumps to 40%, while the occurrence of mixed inhibition cases with *K_ic_>K_iu_* drops to a mere 5%. **Interesting to note is that 66% of the 467 *K_ic_*-*K_iu_* couples were relative to multi-substrate enzymes.**

### Investigation into the mixed inhibition-mimicking mechanisms

In this section, we sought to demonstrate whether MIM mechanisms cause mixed inhibition with *K_ic_*s that are necessarily always less than, or equal to, *K_iu_*s. We first derived a mathematical relation between *K_ic_* and *K_iu_* for each of the five mechanisms (for the sake of brevity this part was moved in the Supplemental Material). Then, to verify the correctness of the derived equations, MIM mechanisms were simulated using KinTek Explorer^36^^,^^37^. To determine *K_ic_*s and *K_iu_*s, as in a real experiment, the computed initial velocities were analysed by the conventional double-reciprocal plot of 1/V vs 1/[S].

#### Case I – tight-binding inhibitors

To determine the *K_i_* of an inhibitor, reactions must be measured in the presence of inhibitor concentrations close to the *K_i_*. This implies that for very potent inhibitors, typically with *K_i_* in the nM range or less, the inhibitor might turn out to be not in concentration higher enough than the enzyme, hence violating the first of the two steady-state assumptions. Methods to analyse tight inhibition have been proposed^41^^,^^42^, but if this circumstance is overlooked and kinetic data are analysed according to the classic steady-state method, active-site directed inhibitors can easily be mistaken for mixed-type inhibitors^43^^,^^44^.

**Figure 3.**
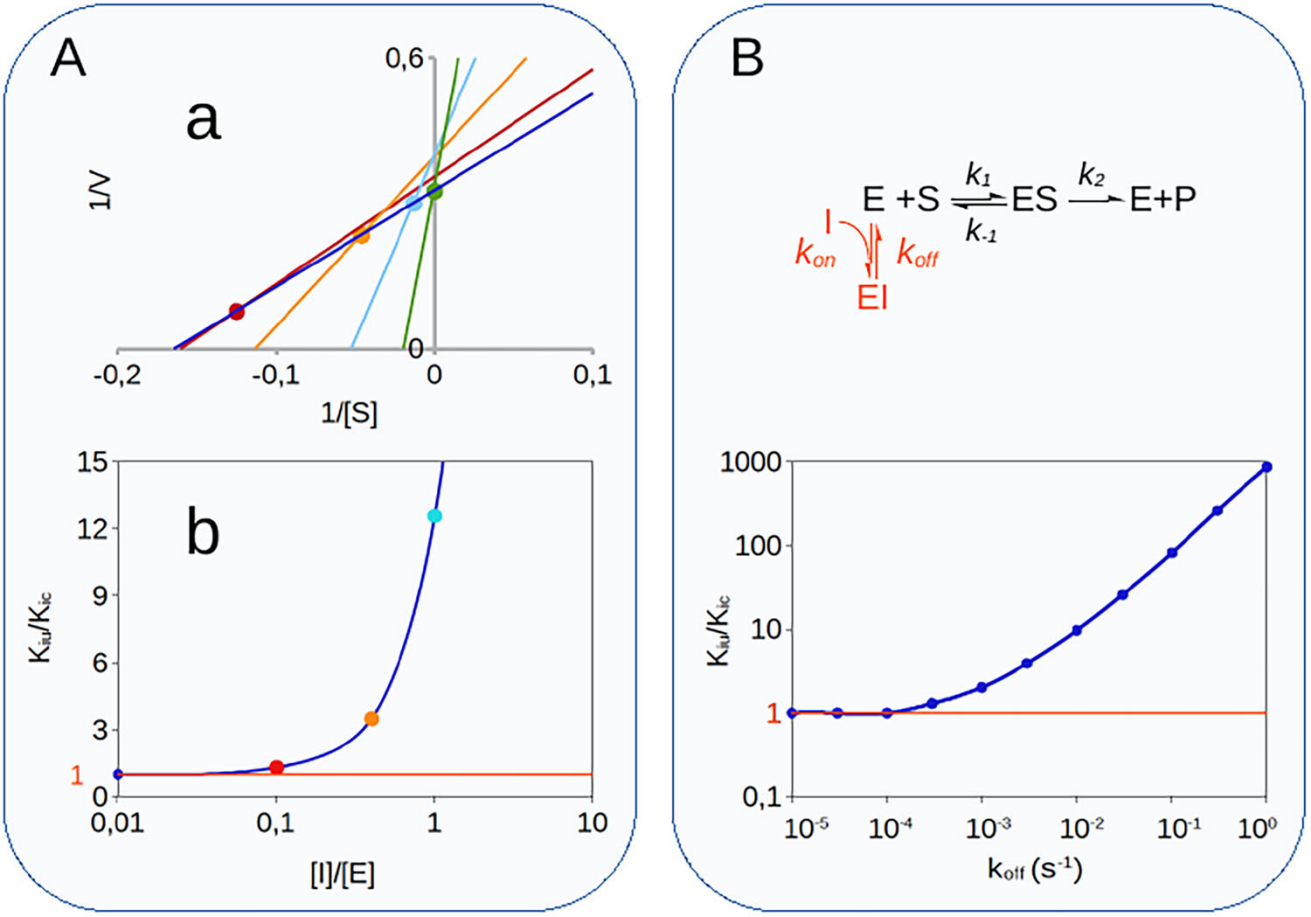
Simulation of MIM mechanisms of type 1 and 2. **(A)**. Tight inhibition. (**a**) Double reciprocal plot of the uninhibited reaction (blue trace) and of reactions inhibited by four competitive inhibitors with different *K_i_* and at different concentrations so that the [I]/*K_i_* ratio is the same for all reactions. As expected for an active site-bound inhibitor, as long as the inhibitor concentration is at least ∼10 times larger than the enzyme concentration (for green trace [I]/[E] = 10), the inhibition looks competitive. If [I] drops below 10·[E], the intercept shifts towards the first quadrant of the plot, mimicking a mixed-type inhibition whose *K_iu_*/*K_ic_* ratio asymptotically tends to 1 as the inhibitor-to-enzyme ratio decreases (b). (**B**). Slow-dissociating inhibitors. For reactions initiated after a long enzyme-inhibitor incubation, the observed initial velocities depend critically on the dissociation rate constant of the inhibitor (*k_off_*). In the presence of active site-bound inhibitors, this mechanism generates an apparent mixed inhibition with *K_iu_*/*K_ic,_* which asymptotically tends to 1 as the dissociation slows.

Figure 3(A) shows the inhibition caused by competitive inhibitors of different potencies supplied at concentrations between 10·[E] and 0.1·[E]. In the simulation, for all inhibitors, the [I]/*K_i_* ratio was set to 10. Therefore, in principle, for all of the reactions, the degree of inhibition should be the same. However, as the [I]/[E] ratio drops below 10, the inhibitor depletion that occurs upon E-I binding can no longer be neglected, and the fraction of the inactive inhibitor-bound enzyme starts to deviate from the expected linear behaviour. In the double reciprocal plot, this is visualised as both a slope and an intercept effect that shifts the point of line intersection towards the first quadrant, thus mimicking a mixed inhibition pattern. The apparent *K_iu_*, which evidently emerges as a mere artefact, only depends on the [I]/[E] ratio: the smaller the ratio is, the smaller the *K_iu_*. As [I] tends to 0, however, the inhibition fades off, and the trace of the inhibited reaction tends to overlap with that of the uninhibited reaction. Therefore, under no circumstance, the intersection point can be below the 1/[S] axis, and hence, the apparent *K_iu_* turns out to be necessarily greater than, or at best equal to, *K_ic_*.

#### Case II – time-dependent inhibition

The steady-state model assumes that the initial velocities are a direct measure of the steady-state rates. In the case of slow-binding inhibitors, however, the onset of the inhibition is delayed, with the consequence that initial velocities underestimate the actual inhibition^45^. A common strategy to tackle slow inhibition is to preincubate the enzyme and inhibitor and to add the substrate as the last reactant so that the enzyme-inhibitor equilibrium is attained before the reaction is started. In this case, however, if the enzyme-inhibitor dissociation is slow, the initial velocities underestimate the steady-state turnover, and the conventional steady-state analysis causes competitive inhibitors to be mistaken for mixed-type or non-competitive. Although the effects of slow inhibitor dissociation on substrate-initiated reactions have long been described^45^, the mechanism leading to the emergence of an apparent uncompetitive inhibition component, to the best of our knowledge, has explicitly been elucidated only very recently^46^. As in all cases where the steady-state assumptions are not met, the output of the conventional data analysis is largely dependent on the experimental setup. In this specific case, the extent of the fictitious uncompetitive component strictly depends on the enzyme-inhibitor dissociation rate and on the dead time for the detection of initial velocities. Readers interested in deepening the mechanistic aspect of this kinetic artefact are referred to the cited literature^46^. Here, it suffices to state that the apparent *K_iu_* asymptotically tends to *K_ic_* as the enzyme-inhibitor dissociation gets slower, eventually mimicking pure non-competitive inhibition (*K_ic_*=*K_iu_*) when *k_off_* is less than ∼1 0 ^−3^–1 0 ^−4^ s ^−1^ (Figure 3(B)), so that necessarily *K_iu_*≥*K_ic_*.

#### Case III – Multisubstrate reactions

The Michaelis–Menten model was derived for single substrate reactions, although these are quite rare in biochemistry and are confined to a few isomerizations. It has been evaluated that some 60% of all the known enzyme-catalyzed reactions involve the conversion of two substrates into two products according to the equation A + B = P + Q^6^. For such reactions, referred to as Bi-Bi reactions, there are three common mechanisms: the substituted-enzyme (or ping-pong) mechanism and the ternary-complex mechanism, either with compulsory or random order of substrate binding. Depending on the actual mechanism, the steady-state analysis of a dead-end inhibitor directed against the binding site of substrate A and assayed against substrate B (i.e. at varying B concentrations and at fixed A) can give mixed-type, pure non-competitive or uncompetitive inhibition. This is a very well-established fact^47–49^ that likely constitutes the most common source of **non** competitive inhibition. Here, we sought to demonstrate whether in this mixed inhibition case too, the uncompetitive inhibition constant, $K_{IU(B)}^{\mathrm{app}},$ is necessarily always greater than or equal to the competitive constant, $K_{IC(B)}^{\mathrm{app}}.$

**Scheme 1.**
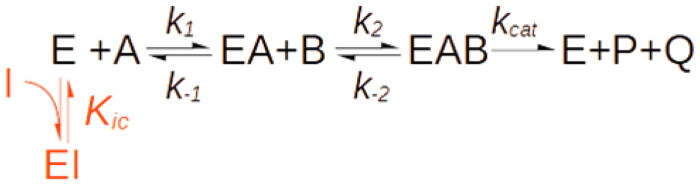
Mechanism of ternary-complex reaction with compulsory order.

**Figure 4.**
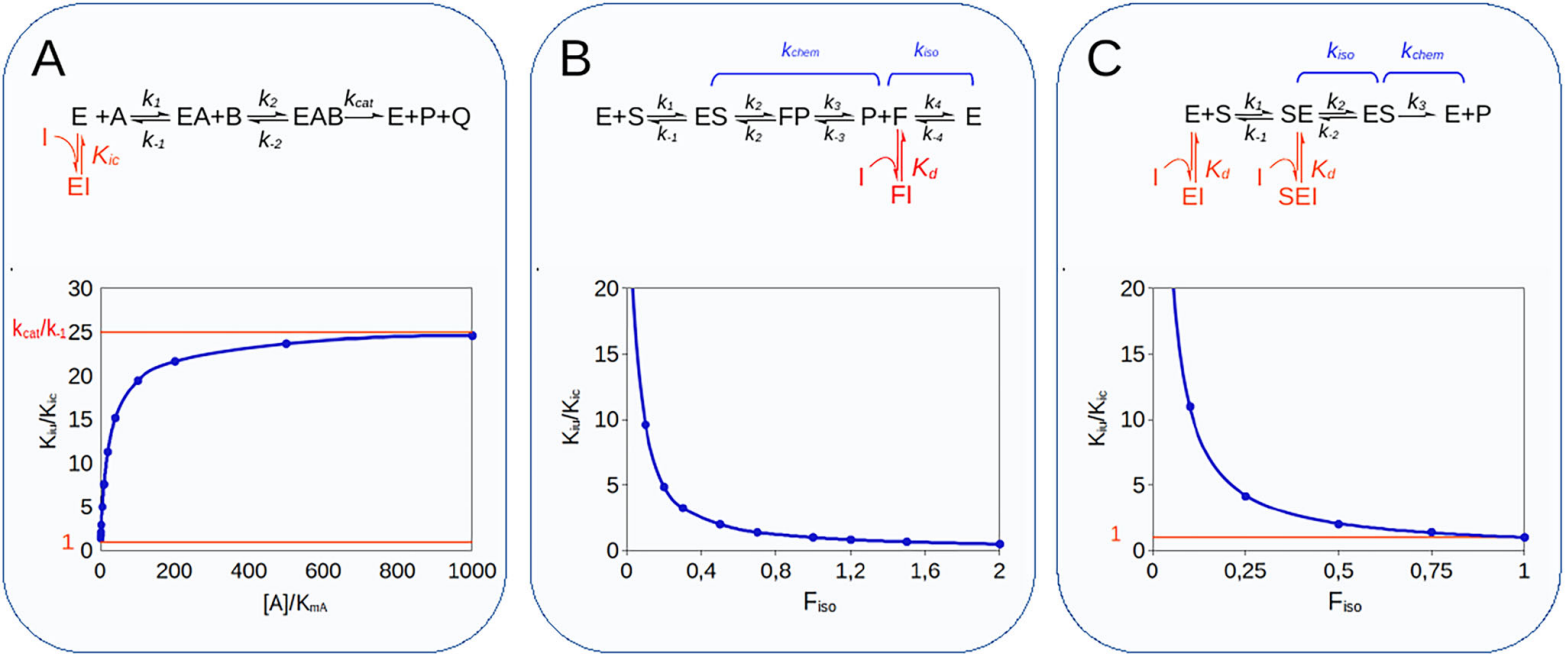
Simulation of MIM mechanisms of type 3, 4 and 5. **(A).** Multisubstrate reactions. For bi-bi reactions with a sequential ordered mechanism, competitive inhibitors directed against the binding site of substrate A, when B is the variable substrate, generate an apparent mixed inhibition with a *K_iu_*/*K_ic_* ratio that tends asymptotically to 1 when [A]/*k_mA_* tends to 0 and to *k_cat_*/*k_-1_* when [A] tends to ∞. **(B)**. For iso-mechanism enzymes, inhibitors that target the product-binding isomer of the free enzyme (F in the reaction scheme) generate mixed-type inhibition with a *K_iu_*/*K_ic_* ratio equal to 1/F_iso_. **(C)**. With exo-site enzymes, active-site directed inhibitors cause mixed inhibition with *K_iu_*/*K_ic_* that tends asymptotically to 1 as F_iso_ tends to 1.

For a ternary-complex reaction with compulsory order of binding, as depicted in Scheme 1, it can be shown (see Supplemental Material) that $K_{IC(B)}^{\mathrm{app}}$ and $K_{IU(B)}^{\mathrm{app}}$ depend on the concentration of substrate A (i.e. the constant substrate) according to the following rules:

$$K_{IC(B)}^{\mathrm{app}}=K_{ic}+\frac{\left[A\right]}{K_{s}}K_{ic}\;$$

$$K_{IU(B)}^{\mathrm{app}}=K_{ic}+\frac{\left[A\right]}{K_{M(A)}}K_{ic}\;$$

where *K_ic_* is the true dissociation constant for the enzyme-inhibitor complex (i.e. the competitive inhibition constant measured when substrate B is constant and A is variable), *K_s_* is the dissociation constant of the enzyme-substrate A complex and *K_M(A)_* is the Michaelis constant for substrate A. At any given concentration of substrate A, the ratio between the apparent uncompetitive and competitive components is:

$$\frac{K_{IU(B)}^{\mathrm{app}}}{K_{IC(B)}^{\mathrm{app}}}=\frac{K_{ic}+\frac{\left[A\right]}{K_{M(A)}}K_{ic}}{K_{ic}+\frac{\left[A\right]}{K_{S}}K_{ic}}$$

which asymptotically tends to 1 as the concentration of substrate A tends to 0 and to *K_s_*/*K_M(A_*_)_ as [A] tends to infinity. This $K_{IU(B)}^{\mathrm{app}}/K_{IC(B)}^{\mathrm{app}}$ ratio dependence on [A] was confirmed by steady-state analysis of the KinTek Explorer simulations (Figure 4(A)). By definition, *K_s_*/*K_M(A_*_)_ is equal to *k_cat_*/*k_-1_*, which for a compulsory order mechanism is always greater than 1 (see Supplemental Material), hence leading to the conclusion that in this type of mixed inhibition, the uncompetitive component is always necessarily greater than the competitive component ($K_{IU(B)}^{\mathrm{app}}$>$K_{IC(B)}^{\mathrm{app}}$).

For ternary-complex mechanisms with random-order, the two inhibition components are identical, and it can be demonstrated that they are equal to:

$$K_{IC(B)}^{\mathrm{app}}=K_{IU(B)}^{\mathrm{app}}=K_{ic}+\frac{\left[A\right]}{K_{s}}K_{ic}$$

Therefore, the $K_{IU(B)}^{\mathrm{app}}$/$K_{IC(B)}^{\mathrm{app}}$ ratio is always equal to 1, and the apparent inhibition against substrate B always appears to be of the pure non-competitive type.

#### Case IV – iso-mechanisms

**Scheme 2.**
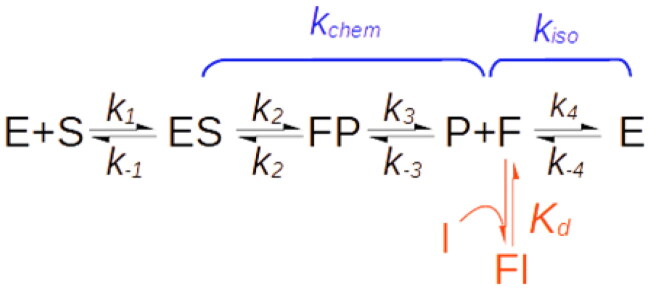
Reaction scheme of iso-mechanism enzymes.

The cyclic nature of enzymatic catalysis implies that once the reaction products are released, the enzyme reverts to the form competent for the binding of the substrate. The Michaelis–Menten model assumes that the isomerisation between the substrate form and the product form (F in Scheme 2) of the enzyme is instantaneous and therefore can be neglected. This, indeed, is true in most cases. There are, however, exceptions, such as isomerases that assume different conformations to bind different isomers, enzymes engaging in general acid or base catalysis that need reprotonation, or hydrolytic enzymes that require rehydratation^50^^,^^51^. If the isomerisation step that restores the initial enzyme form is slow enough to affect catalytic turnover, the enzyme is said to have an iso-mechanism (Scheme 2) ^52^.

Compelling analytical tools to investigate iso-mechanisms were developed only in the 1990s, mainly by Dexter Northrop, who exploited the distinctive inhibition pattern of these enzymes. In the absence of an iso-mechanism, the reaction product is typically a competitive inhibitor of the substrate because both reactants bind the same form of enzyme. In contrast, for enzymes with iso-mechanisms, the substrate and product bind two distinct enzyme forms; therefore, the product inhibition pattern is of the mixed-type^50^^,^^53^^,^^54^. Analogous considerations apply to the case of dead-end inhibitors that exclusively target the product form of the active site.

As shown in Scheme 2, for iso-mechanisms, the overall turnover rate, (*k_cat_*), is made up of two segments, the catalytic step governed by the net rate constant *k_chem_* and the isomerisation step, with net rate constant k_iso_. The binding of an inhibitor to the product form of the enzyme (F in Scheme 2) causes a mixed-type inhibition with:

$$K_{ic}=k_{d}\frac{k_{4}+k_{-4}}{k_{4}}$$

$$K_{iu}=k_{d}\frac{k_{\mathrm{iso}}}{k_{\mathrm{cat}}}$$

with *K_d_* representing the dissociation constant of the FI complex (see Supplemental Material). As correctly predicted by Northrop^50^^,^^51^ under the initial velocity condition, the *K_ic_*/*K_iu_* ratio equals F_iso_ (Figure 4(B)), a parameter that quantifies the kinetic significance of the isomerisation segment and is defined as:

$$F_{\mathrm{iso}}=\frac{\frac{1}{k_{\mathrm{Fiso}}}+\frac{1}{k_{\mathrm{Riso}}}}{\frac{1}{k_{\mathrm{cat}}}}$$

where *k_Fiso_* and *k_Riso_* are the rate constants for the forward and reverse isomerisation steps, respectively.

Unlike the previous MIM mechanisms, in this case, the reduction in the turnover rate caused by active site-bound inhibitors is not just an artefact. Because F_iso_ depicts an intrinsic feature of the catalytic mechanism, all inhibitors of a given enzyme would cause a mixed inhibition with an identical *K_iu_*/*K_ic_* ratio.

The acetylcholinesterase inhibitors developed as potential anti-Alzheimer’s drugs perhaps constitute the most prominent example of mixed dead-end inhibition of an iso-mechanism enzyme^55^. However, there are no reliable data on the prevalence of iso-mechanisms, so it is difficult to estimate their contribution to the overall occurrence of mixed inhibition. It is worth noting that the product form of the free enzyme is a short-lived molecular species generated along with the catalysis rather than a stable enzyme isoform. This implies that isomerisation in the forward direction (F→E) is necessarily faster than in the reverse direction (F←E) ^52^, which is consistent with the finding that the chemical segment is always the rate-limiting step^50^. Consequently, even though the *K_iu_*s generated by iso-mechanisms cannot be algebraically shown to be necessarily greater than the *K_ic_*s, mechanistic considerations explain why F_iso_ is practically always less than 1 and, consequently, why, in this case too, the mixed inhibition is dominated by the competitive component.

#### Case V – exo-sites

With most enzymes, it is not possible to make a clear distinction between the portion of the active site involved in the binding of the substrate and the actual catalytic site because the catalytic site also contributes to substrate stabilisation, at least to some extent. In this respect, enzymes that catalyse the covalent modification of large macromolecular substrates as endonucleases^56^, protein kinases/phosphatases^57^, proteases^58^ and in general, all post-translational modification enzymes, represent a notable exception. Often, this type of enzyme derives most of the affinity for their substrates from a region of the protein surface that is distinct and remote from the catalytic site. The substrate recognition site, in this case, is called an exo-site^58^.

**Scheme 3.**
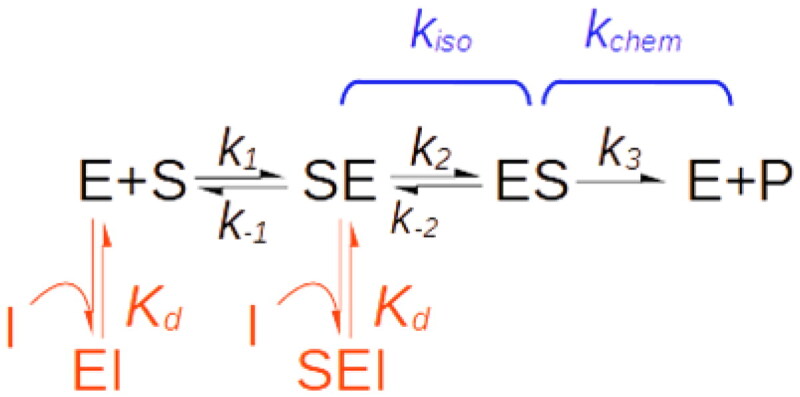
Reaction scheme of exo-site enzymes.

For exo-site enzymes, the binding of the substrate typically proceeds through two sequential steps (Scheme 3), the first leading to an “encounter complex”, here denoted as “SE”, with the substrate bound only to the exo-site. SE then decays monomolecularly to form the catalytically competent complex “ES”, with the consensus sequence of the substrate accommodated within the active site and ready to undergo catalytic conversion.

An active site-directed inhibitor, I, can bind both the free enzyme E and the encounter complex SE but not ES. In other words, the inhibitor does not compete with the initial enzyme-substrate recognition but impedes the isomerisation of the SE complex which leads to the formation of the catalytically competent complex ES. This causes a reduction in the “degree of competition” that in a double reciprocal plot is visualised as a mixed-type inhibition pattern^3^.

To model this reaction mechanism, it is convenient to regard *k_cat_* as composed of two segments, the isomerisation of the SE complex and the chemical catalytic step, governed by the constant rates *k_iso_* and *k_chem_*, respectively (Scheme 3).

Analogous to the previous iso-mechanism case, the coefficient F_iso_, here defined as *k_cat_*/*k_iso_*, quantifies the kinetic relevance of the isomerisation step. F_iso_ assumes values between 0 when *k_iso_* is so large that the isomerisation is kinetically irrelevant and 1 when it is slow enough to be completely rate-limiting.

Simulation of this reaction with KinTek Explorer (Figure 4(C)) showed that the *K_iu_*/*K_ic_* ratio is equal to 1/F_iso_.

From which follows that if F_iso_ tends to 0, the inhibition is competitive, as expected for an active site-bound inhibitor. If F_iso_=1, the catalytic segment of the reaction can be neglected, and the inhibition turns pure non-competitive (*K_ic_*=*K_iu_*). For 0 < F_kiso_<1, the inhibition is of the mixed-type with *K_ic_*<*K_iu_*, so that necessarily *K_iu_*≥*K_ic_* (see Supplemental Material).

The reduction of the affinity for the substrate and of the apparent maximal velocity are both actual. However, as for the previous iso-mechanism case, there is no allosteric site involved, and neither *K_ic_* or *K_iu_* are true dissociation constants.

Although enzymes with exosites are often of utmost pharmacological relevance^3^^,^^59^, the proper study of their inhibition mechanism, which necessarily has to be performed using physiologic substrates, has been limited by the remarkable effort required to produce enough proteinaceous substrates and to measure the time course of their chemical modification. Indeed, this type of study has been reported for just a handful of enzymes, mostly serine proteases of the coagulation cascade process^58^^,^^60^^,^^61^, so that experimental evidence is scant and the contribution of exo-site enzyme inhibition to the statistics of chapter 3 is negligible.

## Discussion

While the definition given by ChatGPT of mixed inhibition is incorrect, it is easy to agree with the concluding remark: “*Understanding the nature of mixed-type inhibition is crucial for studying enzyme kinetics, drug discovery, and the design of effective enzyme inhibitors”.*

The scouting of scientific literature, however, and, ironically enough, the ChatGPT response itself, clearly showed that the proper understanding of mixed inhibition is not something that should be taken for granted. The intrinsic complexity of this type of reversible inhibition made it difficult to devise a clear schematic mechanistic explanation. In turn, this has resulted in descriptions that are often oversimplifying, incomplete and ultimately, misleading. Moreover, to worsen the problem, the great diversity typically displayed by enzymes has discouraged authors from making definitive assertions on the mechanistic irrelevance of the two-site model. With the overall outcome that many of those who deal with the study of enzyme inhibition, perhaps the majority of them, developed an incorrect understanding of the causative molecular determinants of mixed inhibition.

The scope of this work was to assess the actual mechanistic significance of the formal two-site model. The finding that five MIM mechanisms exist that generate mixed inhibition patterns with *K_iu_*s that are inevitably always larger than or equal to their cognate *K_ic_*s, combined with the evidence that in the reported cases of mixed inhibition practically never is *K_ic_*>*K_iu_*, constitutes solid statistical evidence that these MIM mechanisms constitute the actual underlying mechanisms of mixed inhibition.

With the understanding that a molecular mechanism can be established only on the basis of a thorough specific study, the analysis presented herein shows that the two-site formal mechanism substantially plays no role in the generation of mixed inhibition and hence, that the molecular nature of reversible enzyme inhibition is basically just competitive.

This also explains the lack of structural evidence for the binding of mixed inhibitors to allosteric sites: on the basis of the statistic on enzyme inhibition reported in the result section, the Protein Data Bank^62^ should contain at least 4–5000 structures of enzymes in complex with mixed or non-competitive inhibitors. However, in an attempt to assess the occurrence, among mixed inhibitors, of ES complex binders, we found that none of the 197 entries that currently contain the term “noncompetitive inhibitor” or “mixed-type inhibitor” bound sites other than the enzyme active site.

In the first three cases discussed in the results section, i.e. tight inhibition, slow dissociating inhibitors and multiple-substrate enzymes, the inhibition is only apparently of the mixed type: in an *in vivo* scenario, a dead end inhibitor acting through these mechanisms, as for example a drug, would simply cause an inhibition of the competitive type that can be overcome by high substrate concentrations. Regardless, multiple-substrate enzymes remain the most frequent cause of the reported mixed inhibition, as correctly suggested by Johnson^7^. It is worth noting that because highly potent inhibitors are by necessity tight and/or slow-dissociating binders, the MIM mechanism of type I and II might be of special importance in drug design.

In cases IV and V (i.e. iso-mechanism and exo-site enzymes) although the inhibitors only bind to the same site bound by the substrate, the inhibition actually affects both the enzyme’s substrate affinity and limiting velocity and cannot be “resolved” by higher substrate concentrations. As correctly pointed out by Cornish-Bowden^12^, iso-mechanism enzymes likely constitute the main source of biologically relevant mixed inhibition. It should be stressed, however, that the real incidence of mixed inhibition of exo-site enzymes is certainly largely underestimated.

Perhaps, the most convenient approach to disentangle the complexity of mixed inhibition is to dismiss the classic analysis if initial rates in favour of the more up-to-date computer assisted global fitting of full reaction time-courses implemented by software such as KinTek Explorer^36^^,^^37^ or ENZO^63^. The fitting of kinetic data based on numerical integration of rate equations streamlines the otherwise difficult study of mechanisms that deviate from the steady-state assumptions, as are the cases, quite common in pharmacology, of tight-binders and time-dependent inhibitors. In general, once the mechanism of the target enzyme has been resolved, the direct simulation of enzymatic reactions allow to effortlessly distinguish between the five MIM mechanisms.

## Supplementary Material

Supplemental Material
