# Supplementary material for: Mixed and non-competitive enzyme inhibition: underlying mechanisms and mechanistic irrelevance of the formal two-site model
Source: J Enzyme Inhib Med Chem. 2023 Aug 14;38(1):2245168. doi: 10.1080/14756366.2023.2245168 (PMC10683834; doi:10.1080/14756366.2023.2245168)
Supplement: Supplemental Material [file IENZ_A_2245168_SM0998.pdf]

Mixed and noncompetitive enzyme inhibition: underlying mechanisms and mechanistic irrelevance of the formal two-site model

Alessandro Pesaresi[a]*

[a]*Istituto di Cristallografia – Consiglio Nazionale delle Ricerche, Area Science Park, Basovizza, Trieste, I-34149, Italy*

*alessandro.pesaresi@ic.cnr.it

Derivation of the K_{iu}-K_{ic} relation

Case III – Multisubstrate reaction

Sequential reaction with compulsory order

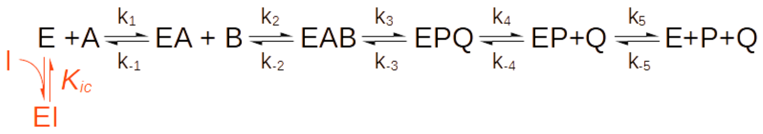

Scheme 1

For a Bi-Bi reaction with a sequential ordered mechanism as depicted in Scheme 1, the equation for the initial rate is:

$$v = \frac{V[A][B]}{K_S K_B + K_B[A] + K_A[B] + [A][B]} \quad \text{(Eq. 1);}$$

where V is the limiting reaction velocity at saturating A and B substrate concentrations, K_A and K_B are the Michaelis constants for A and B, respectively, and K_S is the dissociation constant for substrate A, equal to k_{-1}/k_1. At a fixed substrate A concentration, the apparent maximal velocity (V_B^{app}) observed at saturating B and the apparent Michaelis constant for substrate B (K_B^{app}) are both a function of the concentration of A:

$$V_B^{app} = V\frac{[A]}{[A]+K_A} \quad \text{(Eq. 2),}$$

$$K_B^{app} = \frac{K_S K_B + K_B[A]}{K_A + [A]} \quad \text{(Eq. 3).}$$

In the presence of binding site A-directed inhibitor I, the apparent Michaelian parameters are:

$$V_{B,I}^{app} = V \frac{[A]}{[A]+K_A\left(1+\frac{[I]}{K_{ic}}\right)} \quad \text{(Eq. 4)}$$

$$K_{B,I}^{app} = \frac{K_{s,I}^{app}\ K_B+K_B[A]}{K_A+[A]} \quad \text{(Eq. 5)}$$

where $K_{s,I}^{app}$ is the apparent substrate A dissociation constant observed in the presence of I:

$$K_{s,I}^{app} = K_s(1+\frac{[I]}{K_{ic}}).$$

By definition, mixed inhibition affects both the V/K_M ratio and V, so that:

$$\frac{V^{app}}{K_m^{app}} = \frac{V}{K_m}\left(1+\frac{[I]}{K_{IC}}\right) \quad \text{(Eq. 6)}$$

$$V^{app} = V\ (1+\frac{[I]}{K_{IU}}) \quad \text{(Eq. 7)}$$

where K_{IC} and K_{IU} are the true competitive and uncompetitive inhibition constants, respectively.

To derive the relation between the true dissociation constant of inhibitor I (K_{ic}) and the apparent K_{ic} and K_{iu} (K_{IC}^{app} and K_{IU}^{app}) observed when the inhibitor is assayed at constant A against substrate B, the equations for the apparent V/V^{app} and $V/K_M/(V/K_M)^{app}$ (i.e., Eq.6 and Eq.7) were equated to Eq.4/2 and Eq.5/3.

Derivation of the K_{ic}-K_{IC}^{app} relation:

For the reaction of Scheme 1, at constant A and variable B, V/K_m is:

$$\frac{V_B^{app}}{K_B^{app}} = \frac{Eq.2}{Eq.3} = V\ [A]\frac{1}{K_B(K_s+[A])} \quad \text{(Eq. 8);}$$

and, in the presence of the competitive inhibitor I, $(V/K_M)^{app}$ is:

$$\frac{V_{B,I}^{app}}{K_{B,I}^{app}} = \frac{Eq.4}{Eq.5} = V\ [A]\ \frac{1}{K_B\left(K_{s,I}^{app}+[A]\right)} \quad \text{(Eq. 9);}$$

Therefore, the ratio between V/K_M and $(V/K_M)^{app}$ is:

$$\frac{V_B^{app}/K_B^{app}}{V_{B,I}^{app}/K_{B,I}^{app}} = \frac{Eq.8}{Eq.9} = \frac{K_{S,I}^{app}+[A]}{K_S+[A]} = 1+\frac{[I]}{K_{IC}^{app}} \quad \text{(Eq. 10);}$$

where K_{IC}^{app} represents the apparent competitive inhibition constant against substrate B caused at a fixed A concentration by the binding site A–directed inhibitor I.

From equation 10 follows that:

$$\frac{[I]}{K_{IC}^{app}} = \frac{K_{S,I}^{app} - K_S}{K_S + [A]} = \frac{K_S\left(1 + \frac{[I]}{K_{ic}}\right) - K_S}{K_S + [A]} \text{ (Eq. 11);}$$

and hence that:

$$\boldsymbol{K_{IC}^{app} = K_{ic} + \frac{[A]}{K_s} K_{ic}} \text{ (Eq. 12)}$$

Derivation of the K_{ic}-K_{IU}^{app} relation:

For the reaction of Scheme 1, at constant A and variable B, *V* is V_B^{app} (see Eq. 2), and in the presence of inhibitor I, V^{app} is $V_{B,I}^{app}$(see Eq.

$$\frac{V_B^{app}}{V_{B,I}^{app}} = \frac{Eq.2}{Eq.4} = \frac{[A] + K_A(1 + \frac{[I]}{K_{ic}})}{[A] + K_A} = 1 + \frac{[I]}{K_{IU}^{app}} \text{ (Eq. 13),}$$

where K_{IU}^{app} is the apparent uncompetitive inhibition constant against substrate B caused at a fixed A concentration by the binding site A – directed inhibitor I. From equation 13 follows that:

$$\frac{[I]}{K_{IU}^{app}} = \frac{K_A \frac{[I]}{K_{ic}}}{[A] + K_A} \text{ (Eq. 14);}$$

Hence,

$$\boldsymbol{K_{IU}^{app} = K_{ic} + \frac{[A]}{K_A} K_{ic}} \text{ (Eq. 15).}$$

In conclusion, binding site A-directed inhibitor I causes an apparent mixed-type inhibition against substrate B characterized by a K_{IU}/K_{IC} ratio that is:

$$\frac{K_{IU}^{app}}{K_{IC}^{app}} = \frac{K_{ic} + \frac{[A]}{K_A} K_{ic}}{K_{ic} + \frac{[A]}{K_S} K_{ic}} \text{ (Eq. 16)}$$

Because $K_A = \frac{k_{cat}}{k_1}$, $K_S = \frac{k_{-1}}{K_1}$ and $\frac{K_A}{K_S} = \frac{k_{cat}}{k_{-1}}$, it follows that:

$$\frac{K_{IU}^{app}}{K_{IC}^{app}} = \frac{K_{ic}+\frac{[A]}{K_A}K_{ic}\frac{k_{-1}}{k_{cat}}}{K_{ic}+\frac{[A]}{K_A}K_{ic}} \text{ (Eq. 17).}$$

From equation 17, it is evident that $K_{IU}^{app}/K_{IC}^{app}$ tends asymptotically to 1 if [A] tends to 0 and to $\frac{k_{-1}}{k_{cat}}$ if [A] tends to infinity. This implies that $K_{IU}^{app} \geq K_{IU}^{app}$ if $k_{-1} \geq k_{cat}$.

Mechanistic considerations suggest that k_{-1} is necessarily larger than k_{cat}:

the overall turnover rate, k_{cat}, results from the combination of the constant rate for the actual catalytic conversion of substrate into products (k_3) and the net rates for the dissociation of the products P and Q (see Scheme 1), according to the equation:

$$k_{cat} = \frac{k_3 k_4 k_5}{k_{-2}+k_3+k_2 k_4+k_2 k_3} \text{ (Eq. 18),}$$

from which it is evident that k_{cat} is necessarily smaller than any of k_3, k_4 or k_5, and specifically that $k_5 >> k_{cat}$.

In enzymes exhibiting a compulsory order mechanism, only the binding site of substrate A is constitutively present on the free enzyme. The binding of A then induces a structural rearrangement that results in the formation of the binding site for substrate B, which explains why B can only bind after A. k_{-1} is the kinetic constant for the reverse of this process, that is, the dissociation of the EA complex and the consequent reversion to the constitutive free enzyme form. This transition is identical to that occurring at the very end of the catalytic turnover, when the product Q is released and the enzyme reverts to the free form E. Hence, because the two microscopic constants k_{-1} and k_5 describe the same process, whose rate limiting step is the protein structural rearrangement, they can be expected to have similar values. Moreover, it should be considered that the rate-limiting step of enzymatic turnover often is the chemical conversion, in this case represented by the constant k_3. So that, in conclusion, since $k_5 >> k_{cat}$ and $k_{-1} \simeq k_5$, follows that $k_{-1} > k_{cat}$, and hence, $K_{IU}^{app} \geq K_{IU}^{app}$.

Sequential reaction with random order

For sequential random-order reactions, the initial velocity equation is:

$$v = V\frac{\frac{[A][B]}{K_{sA}K_B}}{1+\frac{[A]}{K_{sA}}+\frac{[B]}{K_{sB}}+\frac{[A][B]}{K_{sA}K_B}} \text{ (Eq. 19);}$$

where K_{sA} and K_{sB} are the dissociation constants for substrates A and B, respectively.

At a fixed A concentration, the apparent maximal velocity measured at saturating B and the apparent Michaelis constant for B are:

$$V_B^{app} = V\frac{[A]}{[A]+K_{sA}} \text{ (Eq. 20)}$$

$$K_B^{app} = K_B \text{ (Eq. 21).}$$

In the presence of the substrate A binding site-directed inhibitor I, the apparent Michaelian parameters are:

$$V_{B,I}^{app} = V\frac{[A]}{[A]+K_{sA}(1+\frac{[I]}{K_{ic}})} \text{ (Eq. 22)}$$

$$K_{B,I}^{app} = K_B \text{ (Eq. 23).}$$

Derivation of the K_{ic}-K_{IC}^{app} relation:

For the reaction of Scheme 1, at constant A and variable B, V/K_m is:

$$\frac{V_B^{app}}{K_B^{app}} = \frac{Eq.20}{Eq.21} = V[A]\frac{1}{[A]+K_{sA}}\frac{1}{K_B} \text{ (Eq. 24).}$$

The V^{app}/K_m^{app} ratio caused by substrate A binding site-directed inhibitor I is:

$$\frac{V_{B,I}^{app}}{K_{B,I}app} = \frac{Eq.22}{Eq.23} = V[A]\frac{1}{A+K_{sA}(1+\frac{[I]}{K_{ic}})}\frac{1}{K_B} \text{ (Eq. 25).}$$

Thus, the ratio between V/K_m and $(V/K_m)^{app}$ is:

$$\frac{V_B^{app}/K_B^{app}}{V_{B,I}^{app}/K_{B,I}^{app}} = \frac{Eq.24}{Eq.25} = \frac{[A]+K_{sA}\left(1+\frac{[I]}{K_{ic}}\right)}{[A]+K_{sA}} = 1+\frac{[I]}{K_{IC}^{app}} \text{ (Eq. 26),}$$

where K_{IC}^{app} represents the apparent competitive inhibition constant against substrate B caused at a fixed A concentration by binding site A – directed inhibitor I.

From equation 26 follows that:

$$\frac{[I]}{K_{IC}^{app}} = \frac{K_{sA}\frac{[I]}{K_{ic}}}{[A]+K_{sA}} \text{ (Eq. 27).}$$

Hence,

$$\boldsymbol{K_{IC}^{app} = K_{ic} + \frac{[A]}{K_{sA}} K_{ic}} \text{ (Eq. 28).}$$

Derivation of the K_{ic}-K_{IU}^{app} relation:

For the reaction of Scheme 1, at constant A and variable B, V is V_B^{app} (see Eq. 24), and in the presence of inhibitor I, V^{app} is $V_{B,I}^{app}$(see Eq. 25), so that the V/V^{app} ratio is given by:

$$\frac{V_B^{app}}{V_{B,I}^{app}} = \frac{Eq.20}{Eq.22} = \frac{[A]+K_{sA}(1+\frac{[I]}{K_{ic}})}{[A]+K_{sA}} = 1 + \frac{[I]}{K_{IU}^{app}} \text{ (Eq. 29);}$$

where K_{IU}^{app} represents the apparent uncompetitive inhibition constant against substrate B caused at a fixed A concentration by binding site A – directed inhibitor I.

From equation 29, it follows that:

$$\boldsymbol{K_{IU}^{app} = K_{ic} + \frac{[I]}{K_{sA}} K_{ic}} \text{ (Eq. 30).}$$

Hence, for sequential reactions with random order, K_{IU}^{app} is always equal to K_{IC}^{app}, and consequently, the inhibition results are necessarily pure noncompetitive.

Case IV – Iso-mechanism

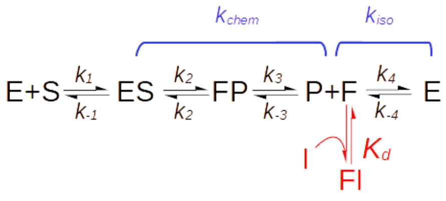

Scheme 2.

In iso-mechanism reactions, the turnover rate is made up by the contribution of two components, the chemical segment, which accounts for the actual catalytic step and for the release of the reaction product, with net rate k_{chem}, and the isomerization segment, which accounts for the conversion of the

enzyme product form (F) into the initial substrate form (E), with net rate k_{iso}. The overall turnover rate, k_{cat}, is given by:

$$k_{cat} = \frac{k_{chem}k_{iso}}{k_{chem}+k_{iso}} \text{ (Eq. 31).}$$

The inhibitor "I", which binds the F form of the enzyme with a dissociation constant equal to K_d, does not affect k_{chem} but reduces the apparent k_{iso} according to:

$$k_{iso}^{app} = k_{iso}\frac{1}{1+\frac{[I]}{K_d}} \text{ (Eq. 32).}$$

Consequently, the apparent k_{cat} observed in the presence of I is:

$$k_{cat}^{app} = \frac{k_{chem}k_{iso}\frac{K_d}{K_d+[I]}}{k_{chem}+k_{iso}\frac{K_d}{K_d+[I]}} \text{ (Eq. 33).}$$

Due to the presence of a fast equilibrium between E and F, the binding of I to F causes a competitive inhibition whose K_{ic} depends both on the K_d and the forward and reverse isomerization rates:

$$\boldsymbol{K_{IC} = K_d\frac{k_4}{k_4+k_{-4}}} \text{ (Eq. 34).}$$

By definition, for uncompetitive inhibition, we have that:

$$\frac{V^{app}}{V} = \frac{k_{cat}^{app}}{k_{cat}} = \frac{1}{1+\frac{[I]}{K_{IU}}} \text{ (Eq. 35),}$$

where K_{IU} is the uncompetitive inhibition constant. To derive the relation between the true dissociation constant (K_d) and the observed uncompetitive inhibition constant (K_{IU}), k_{cat}^{app}/k_{cat} (Eq. 33/Eq. 31) ratio was equated to $\frac{V^{app}}{V}$ (Eq. 35):

$$\frac{k_{cat}^{app}}{k_{cat}} = \frac{k_{chem}k_{iso}\frac{K_d}{K_d+[I]}}{k_{chem}+k_{iso}\frac{K_d}{K_d+[I]}}\,\frac{k_{chem}+k_{iso}}{k_{chem}k_{iso}} = \frac{1}{1+\frac{[I]}{K_{IU}}} \text{ (Eq. 36).}$$

Form equation 36, by isolating the K_{iu} and K_d terms, follows that under the initial velocity assumption:

$$\boldsymbol{K_{IU} = K_d\frac{k_{chem}+k_{iso}}{k_{chem}} = K_d\frac{k_{iso}}{k_{cat}}} \text{ (Eq. 37).}$$

Case V – Exo-site

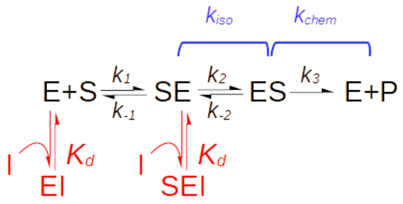

Scheme 3.

Scheme 3 depicts the mechanism of an exo-site enzyme. Similar to the previous Iso-mehanism case, the turnover rate can be regarded as made up of two contributions, the isomerization of the encounter complex (SE) to form the catalytically competent complex (ES), with net rate k_{iso}, and the actual catalytic step, with net rate k_{chem}, so that the overall turnover rate (k_{cat}) is given by:

$k_{cat} = \frac{k_{iso}k_{chem}}{k_{iso}+k_{chem}}$ (Eq. 31).

If we assume that the exo-site and active site are completely independent and hence that the interaction of the substrate with the exo-site does not affect the binding of the inhibitor, the K_{ic} determined by steady-state analysis corresponds to the actual EI (or SEI) dissociation constant (K_d) independent of the kinetic relevance of the isomerization step. Hence:

$\boldsymbol{K_{IC} = K_d}$ (Eq. 39).

The binding of the active-site directed inhibitor I to the ES complex affects the apparent k_{iso} so that:

$k_{iso}^{app} = \frac{k_{iso}1}{1+\frac{[I]}{k_d}}$ (Eq. 32).

Consequently, the apparent k_{cat} observed in the presence of I is:

$k_{cat}^{app} = \frac{k_{chem}k_{iso}\frac{K_d}{K_d+[I]}}{k_{chem}+k_{iso}\frac{K_d}{K_d+[I]}}$ (Eq. 33).

From which follows (see previous demonstration) that:

$K_{IU} = K_d\frac{k_{iso}}{k_{cat}}$ (Eq. 37).

The K_{iu}/K_{ic} ratio can be obtained by simply combining equations 39 and 37:

$\boldsymbol{\frac{K_{IU}}{K_{IC}} = \frac{k_{iso}}{k_{cat}}}$ (Eq. 38).
